# Assessing the Oral Microbiome in Women of Reproductive Age: A Narrative Review

**DOI:** 10.3390/clinpract15110206

**Published:** 2025-11-11

**Authors:** Tiberiu H. Ionaș, Mona Ionaș, Radu Chicea, Dragoș A. Dădârlat, Laura Ștef

**Affiliations:** 1 Department of Dental Medicine and Nursing, Faculty of Medicine, Lucian Blaga University of Sibiu, 550169 Sibiu, Romania; 2 Dental Medicine Research Center, Faculty of Medicine, Lucian Blaga University of Sibiu, 550169 Sibiu, Romania; 3 Department of Surgery, Faculty of Medicine, Lucian Blaga University of Sibiu, 550169 Sibiu, Romania

**Keywords:** oral microbiome, menstrual cycle, hormones, 16S rRNA sequencing, metagenomic sequencing

## Abstract

The oral microbiome may be an indicator of oral pathologies and hormonal fluctuations. Consequently, the proper identification of methods for studying microbial factors is essential. Because more than half of the components of the oral microbiome belong to species that are very difficult or even impossible to cultivate in the laboratory, the assessment of the oral microbiome nowadays is based on genetic sequencing, using techniques such as DNA hybridization, 16S rRNA sequencing, and metagenomics, mainly analyzing saliva and subgingival plaque. Variations in results may be caused by differences in sample type, analysis methods, accuracy in determining cycle phases, and biases introduced by DNA extraction techniques and technical variations. Choosing the right primers for the 16S rRNA gene and reference databases (like HOMD, Greengenes2) is essential for accurately identifying microorganisms. Metagenomic sequencing offers greater taxonomic and functional detail, but it is costlier and presents bioinformatics challenges, including contamination with human DNA. When the patients under study are women, we have to take into consideration the cyclical changes in the menstrual cycle. Studies suggest that estrogen influences local immune and inflammatory responses and can worsen existing gingival inflammation. Certain oral bacteria can even utilize estradiol and progesterone as growth factors. The composition of the oral microbiome is also affected by hormonal contraceptives, carbohydrate intake, smoking, age, body mass index, genetics, and oral hygiene—all factors that need to be controlled for in future studies. Interpreting the biological significance of the reported cyclic changes requires careful examination of the specific methods used in each study.

## 1. Introduction

Studies have shown that the oral microbiome of healthy people is clearly different to that of those who have periodontal disease [1]. However, one of the main problems regarding the oral microbiome is the fact that most of the microbes belong to species that cannot be identified through classic cultivation techniques in the laboratory [2]. Therefore, the assessment of the oral microbiome nowadays is based on genetic sequencing.

The relations between gastrointestinal microbiota and diseases are starting to become well established [3]. In the case of the oral microbiome, studies have shown that specific changes occur between a healthy status and periodontal disease; however, there is limited consensus regarding the significance of these changes [1,4,5].

The oral cavity is a unique microbial niche, distinct from the intestinal cavity, that responds directly to hormonal signals [4,6]. The menstrual cycle is a fundamental physiological process that characterizes the reproductive life of women, and is orchestrated by predictable cyclic fluctuations in the sex hormone steroids, mainly estradiol (an estrogen) and progesterone, under the control of the hypothalamic–pituitary–ovarian axis [7]. The direct sensitivity of oral tissues to hormones, combined with regular hormonal fluctuations of the menstrual cycle and clinical observations of hormonally induced oral changes, supports the hypothesis that the oral microbiome also undergoes cyclical changes [8,9]. 

The number of studies focusing on women of reproductive age is relatively limited compared to research focused on pregnancy or menopause, and the results are often inconclusive, reflecting methodological heterogeneity [6,10]. Periodontal diseases are frequently associated with hormonal changes from pregnancy or menopause, but some studies attribute the observed changes to other factors, such as oral hygiene [10,11,12]. The women-specific group of diseases is unfortunately not well-represented enough in the scientific literature [13]. A specific search of the PubMed database, using the keywords of this article, returned no results. Using the same database, we performed a search about the oral microbiome and separately searched for the effect of the menstrual cycle on oral tissues. Then, we further refined the search, excluding articles about cancer and other specific diseases.

## 2. Oral Microbiome Study Techniques

Studies identified in the literature have employed various approaches, ranging from classic deoxyribonucleic acid (DNA) hybridization techniques to 16S gene ribosomal ribonucleic acid (rRNA) sequencing and “shotgun” metagenomics, predominantly analyzing saliva samples, but also subgingival plaque. Differences in the sample type, microbiome analysis method, and accuracy of cycle phase determination can contribute to the variability of results.

Interpreting the results of oral microbiome studies made through genetic testing requires a thorough understanding of potential sources of variation and bias. They can occur at various stages of the experimental and analytical process, significantly altering the study results [14]. Even the operating system of the computer used to run bioinformatic programs can alter the results [15].

Genetic testing of the microbiome remains a challenging process. Each step could introduce errors, rendering the results useless or even harmful [16]. The first step is to obtain microbial DNA, the second step is to prepare the DNA for reading with specific equipment (library preparation), the third step is to use bioinformatics to transform the raw data into genetic sequences that are identified by comparing with genomic databases, and the last step is to create a report about the microbiome using further bioinformatics tools (Table 1: Microbiome 16S rRNA gene testing steps). 

The primary challenge in designing research on the oral microbiome is the selection of the biological sample type (see Table 2). Saliva provides a global picture of the oral microbial community, and is easy to collect non-invasively, but it contains a mixture of microorganisms from various niches [2,17]. Collection methods, e.g., passive spitting vs. oral rinsing, can influence the results [17]. Samples collected from the dorsal side of the tongue, cheeks, and above and below the gingiva are also described. Each collection site has its particularities and influences the results.

In a 2017 study, Kageyama et al. showed that changes in subgingival plaque over time can be measured by saliva tests. The structure of the microbiome varies with sample collection location, but the changes over time in the same person are statistically correlated, so that the evolution of periodontal disease can be followed by saliva tests [18]. It should be noted, however, that there is a difference between the study conducted by Kageyama et al. [18], who collected saliva after the patient chewed gum for 5 minutes, and the study by Bang et al. [17], who collected saliva passively or by rinsing with 0.9% saline.

Plaque sampling from a specific site (supra- or subgingival) provides information about niches relevant to diseases such as periodontitis, but it is more invasive and can vary depending on the collection technique. Dental calculus sampling is also mentioned in the scientific literature [16,19].

The next step is to prepare the samples for storage. In some studies, the DNA extraction method is started immediately after the sample collection. In most cases, the researchers do not use the samples directly, and some preprocessing treatments are performed. For example, samples of the gut microbiome may be kept in vials with preservatives at room temperature, e.g., OMNIgene (DNA Genotek Inc., Ottawa, ON, Canada) or DNA/RNA Shield (Zymo Research Corp., Irvine, CA, USA), or may be frozen at −20/−80 °C. In a paper published in 2022, Furuhashi et al. did not find a difference in microbiota profiles after 16S rRNA V1–V2 region sequencing between the immediate extraction and three storage conditions [20].

To increase the quality of genetic testing of the microbiome, it is recommended to reduce the quantity of extracellular DNA. Thus, DNA amplification will be directed to the DNA contained in viable cells. In addition, depleting the samples of human viable cells will increase the quality of bacterial DNA measurements [21,22,23,24].

Bacterial DNA extraction methods can introduce significant biases, favoring the lysis of certain types of bacteria (e.g., Gram-positive vs. Gram-negative, *Pseudomonas aeruginosa* (*P. aeruginosa*), *Achromobacter xylosoxidans* (*A. xylosoxidans*)) and thus affecting the relative representation of taxa in the final analysis. It has been observed that even pure microbiological samples do not give the same results if the DNA extraction and preparation technique is changed [22,25,26,27]. Technical variations introduced between different experiments or sequencing rounds, such as different reagents, processing times, operators, and batch effects, can create artificial differences between groups, masking or mimicking real biological effects. Correcting or accounting for these effects is important [15,28,29].

For over 30 years, the testing of the 16S rRNA gene has been used as a standard in microbiological studies. The 16S rRNA gene contains conserved regions as well as variable or hypervariable regions named from V1 to V9, which allow taxonomic differentiation [29,30,31,32]. 

Molecules called primers have also been designed that can connect to the conserved regions and “cut” the 16S gene. The nucleotide sequence for the primer is a marker used by the analysis software to identify the desired readings. The choice of primers that flank variable regions is critical. To be able to differentiate between microbial genera and species, a longer nucleotide read is needed. The 16S gene is around 1550 base pairs (bp) long, while the primers create sections that are between 178 bp and 567 bp (Table 3). Different primers (e.g., targeting V1–V2, V3–V4, V4, V4–V5) have different amplification efficiencies for various taxa, which can distort the relative abundance of detected bacteria [19,33]. Sequencing only a portion of the 16S gene often limits resolution at the genus level, making it difficult to differentiate closely related species [33].

Chakravorty et al. explored the ability of the 16S rRNA gene variable section to identify microbes and concluded that one must choose the primers used in the research according to the details of the targeted microbes. They observed that the V1 region is better at distinguishing between different species of *Staphylococcus*, V2 for *Mycobacterium*, and V3 for *Klebsiella* [34]. Nagai and Shiba et al. attempted to address methodological heterogeneity (16S vs. shotgun, specific primers, bioinformatics pipelines, sample size, accuracy of phase determination) and studied the quality of the 16S gene measurement results starting from samples with known content (microbial cultures). Their study found that at the level of the technologies used, the best results were for specific combinations between the substances used to identify the 16S gene (primers for V1–V2, V3–V4), reference genome databases for comparison (SILVA, Greengenes2, and Human Oral Microbiome Database), and data processing methods [19]. 

Illumina platforms (e.g., MiSeq, NextSeq, Illumina Inc., San Diego, CA, USA [35]) are commonly used for nucleotide sequencing, providing high-accuracy readings, usually of lengths between 2 × 250 bp and 2 × 300 bp [19]. Limiting testing to the 16S gene alone means that the functional potential of the microbial community is only predicted (e.g., by PICRUSt) and not directly measured [36].

In recent years, metagenomic sequencing has been increasingly used; this involves sequencing the entire microbial DNA in a sample, bypassing the specific amplification of the 16S gene. Advantages include the potential to achieve higher taxonomic resolution (down to the species/strain level), the ability to directly analyze the functional potential of the community by identifying metabolic and virulence genes, and reduced polymerase chain reaction (PCR) amplification bias. The major disadvantages are the higher cost, the need for a larger amount of microbial DNA, the challenge posed by significant contamination with human DNA (which can reach 20–95% or even more in oral samples and must be removed bioinformatically), and the increased complexity of bioinformatics analysis [22,37]. Relatively new equipment on the market, like the Minion of Oxford Nanopore Technologies [38], allows for whole genome sequencing at an acceptable price and outside a specialized laboratory environment.

Bioinformatics analysis is a critical step that can significantly influence results. It is necessary to eliminate poor-quality readings, merge paired-end readings (if applicable), and eliminate chimeric sequences (PCR artifacts) [17]. Another problem mentioned in the literature is that microbes can have several identical or slightly different 16S rRNA genes. This can lead to an overestimation of the proportion of bacterial species that have more than 16S copies, ranging between 2 and 11, within the examined microbiome. Another issue related to the number of copies of 16S is their intracellular variability, which, beyond a certain value, can lead to an erroneous classification of the microbes studied [28,29]. 

Traditional approaches group nucleotide sequences into operational taxonomic units (OTUs) based on a similar threshold (usually 97%). Newer methods, such as DADA2 and Deblur, implemented in platforms like QIIME2 and Bioconductor, are based on ampliconic sequence variants (ASVs), which represent unique biological sequences, correcting sequencing errors at the nucleotide level. Although ASVs offer finer resolution, the number of features generated and diversity metrics can differ significantly from OTU methods and even between different ASV methods (e.g., DADA2 vs. Deblur), requiring caution when comparing studies using different approaches [15].

Comparing representative sequences (OTUs) or ASVs with reference databases is crucial for the identification of microorganisms. The choice of database (e.g., SILVA, Greengenes/Greengenes2, RDP, HOMD) can significantly influence the accuracy of the classification. The Human Oral Microbiome Database (HOMD) is specially curated for the oral microbiome and can provide a more accurate classification for oral samples, especially in combination with certain primers (e.g., V1–V2). Greengenes2 also showed good performance in oral studies, especially with regions V3–V4 or V4. Classification algorithms (e.g., RDP Naive Bayesian Classifier, BLAST, VSEARCH/SYNTAX) also have different performance and operating principles [19,29,39].

## 3. Influence of Hormonal Factors on Oral Cavity Tissues and Microbiome

Oral tissues, such as the gums, oral mucosa, and salivary glands, are recognized targets for female sex hormones, expressing specific receptors like the beta estrogen receptor [40]. Studies have shown that these tissues respond to variations in hormone levels, manifesting vascular, inflammatory, and cellular changes during major hormonal events in a woman’s life, such as puberty, pregnancy, and menopause [6,41,42,43,44].

**Figure 1:**
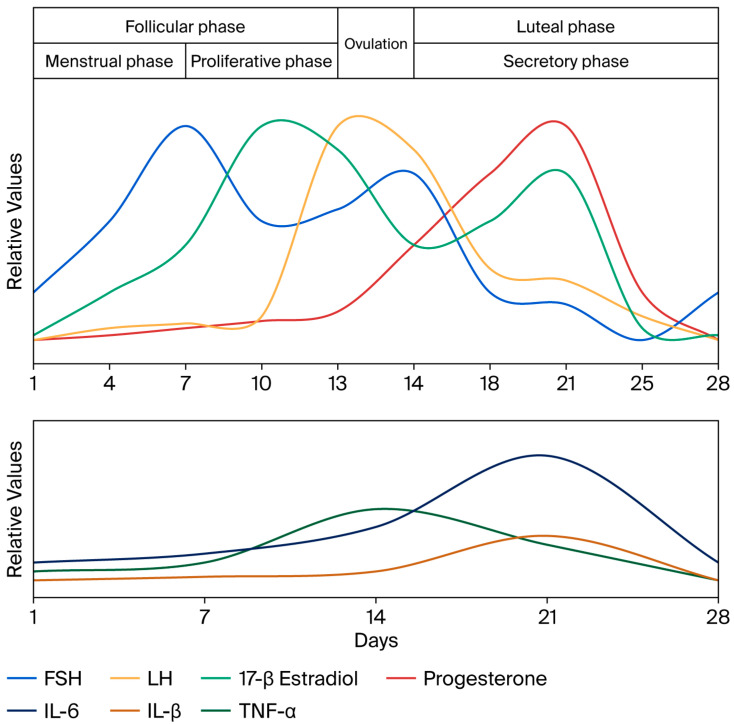
The graphic shows the peaks during the menstrual cycle for follicle-stimulating hormone (FSH), luteinizing hormone (LH), 17-β estradiol, and progesterone, with relative values used as described by Reed and Carr [52]. The lower part shows the relative levels for interleukin-6 (IL-6), interleukin-1β (IL-1β), and tumor necrosis factor-α (TNF-α) as determined by Ndjoh et al. [51] in the gingival crevicular fluid. Data correlated from references [7,51,52,53,54].

Hormonal fluctuations during the menstrual cycle affect oral tissues, which have receptors for female sex hormones and can show vascular, inflammatory, and cellular changes. The oral cavity, being a microbial niche sensitive to hormonal signals, can experience cyclical shifts in its microbiome. Estrogens are modulators of the local immune and inflammatory responses. They inhibit neutrophil chemotaxis by altering neutrophil activity during the normal menstrual cycle and during pregnancy, and may influence immune cell-mediated inflammation [45,46,47,48,49]. Clinically, these influences often translate into an exacerbation of the gingival inflammatory response to existing bacterial plaque at different times, such as during puberty or pregnancy, when taking oral contraceptives, and in the postmenopausal stage [50]. The evolution of hormonal levels during the menstrual cycle is represented in the upper part of Figure 1 and is associated with changes in the gingival tissue [51]. It was demonstrated that in the luteal phase of the menstrual cycle, the quantity of gingival crevicular fluid increases simultaneously with an increase in inflammatory cytokine levels.

Sex hormones can interact directly with certain oral bacteria. It has been shown that *P. intermedia* can use estradiol and progesterone as growth promoters, especially under conditions of vitamin K limitation [55,56]. The growth of *Prevotella* species can thus be favored during periods of high hormone levels [57,58]. 

*Porphyromonas gingivalis* (*P. gingivalis*) can significantly alter the oral tissues (gingiva) and is involved in modulating the inflammatory response of local tissues [59]. Given its ability to simultaneously activate and inhibit pro-inflammatory cytokine production and cellular immunity, it is highly probable that the effect of female hormones on oral tissues can be used to increase colonization at the periodontal level.

On the one hand, hormone levels do not appear to induce gingival changes on their own, but they can alter the responses of periodontal tissue to microbial plaque and thus indirectly contribute to periodontal disease. They play a role in the severity of inflammation [18,60,61]. On the other hand, it has been identified that gingivitis is not consistently accompanied by a proportional increase in the amount of dental plaque, which could support the idea that the host’s inflammatory response to existing plaque is hormonally modulated [62]. This exacerbation of inflammation is likely a direct result of hormonal effects on the vasculature and local immune cells [60,63].

Older studies, conducted when doses of hormones from contraceptives were higher, suggested a link to gingivitis [4], but current evidence indicates a minimal or insignificant impact of modern hormonal contraceptives on the composition or diversity of the oral microbiome, compared to the influence of the endogenous cycle phase [4,64]. However, effects on the gut microbiome have been reported even at these low doses [65].

One can observe an inconsistency in research methodology regarding hormonal level measurements. Female hormonal changes are assessed using a plethora of different techniques. Some researchers use basic methods such as the calendar method (well-established but not very precise), and the alternatives include going up to a high-precision level of measuring in the laboratory, and taking into account the values for follicle-stimulating hormone (FSH), estradiol (E2), luteinizing hormone (LH), and progesterone (PGN) [6,51,61,66]. To detect very subtle effects from an experimental factor, highly precise measurements are essential. The design of a study will establish the required measurement precision needed to establish the scope of the research, but it will also generate limitations that will affect the possibility of comparing results to those of other studies.

Results regarding alpha diversity, the richness and uniformity of species for the same person, are inconsistent. No statistically significant differences were systematically identified between microbial proportions in different phases of the menstrual cycle, regardless of the genetic testing technique used [4,6,62,67]. The observation of high intra-individual stability supports the idea of resilience in the core microbiome at the individual level, suggesting that the differences reported between phases in some studies could be partly methodological artifacts [4,6,65,67].

## 4. Other Important Factors

In addition to methodological variations, the interpretation of changes in the oral microbiome in the context of the menstrual cycle must also account for other factors that can influence microbial composition. 

The consumption of fermentable carbohydrates, especially sugar, is the main factor in the etiology of dental caries and directly influences the composition of the oral microbiome. A link between carbohydrate consumption and microbial variations throughout the menstrual cycle is also mentioned in the literature [4,68]. A major risk factor for periodontal disease is smoking. In addition to the harmful effects on the body, smoking is associated with an increase in the abundance of the genera *Prevotella* and *Veillonella* in saliva [4,69]. Age, body mass index (BMI), host genetic factors, individual oral hygiene, and the presence of other systemic conditions (e.g., diabetes) are known factors that modulate the microbiome [70]. Sex itself is a major determinant of oral microbiome composition [71,72,73]. All these factors must be controlled for or considered in the design and analysis of studies on the influence of the menstrual cycle on the oral microbiome.

## 5. Discussions and Conclusions 

Because dental medicine and general medicine usually follow separate curricula from one point onward, it is difficult for practitioners of one domain to step into the other. As a result, advances in genetics, immunology, microbiome studies, and other domains are reaching dentistry later. As an observation, we have a lot of commercially available tests for all kinds of general medical diseases. Quick tests for viruses (for example, flu, COVID-19) and microbes are available in general practitioners’ offices [74,75,76]. At a higher medical level, one has access to advanced tests, going up to, for example, genetic testing of microbes or tissues presumed to be the cause of the disease [74].

By contrast, in general dentistry, the diagnosis of periodontal disease is mostly made based on clinical observations, helped by an X-ray examination. The etiopathogenesis of periodontal disease is very complex. Therefore, many factors are involved that one can literally find a link to the disease in almost all dental medicine books. Immunity, genetics, hygiene, care, diet, smoking and other unhealthy habits, occlusion, sex, hormones, microbes, viruses, and many more factors contribute to the progression of this disease [77]. The link behind all these factors is tissue inflammation and its subsequent effects. 

While we can discuss genetic inheritance for a periodontal disease patient, our ability to influence this factor has more of a theoretical value at this moment [78]. Hygiene, diet, and healthy habits can be taught, but are dependent on the patient [79]. After the dentist has corrected dental problems like cavities and occlusion, the remaining factors revolve around immunity/inflammation and microbiome [78].

Immunity and inflammation are closely linked and specific to a moment in the life of a patient. General health status, diet, stress level, genetics, hormones, and many more aspects will change how people react to internal and external factors [80,81]. The oral cavity is exposed many times a day to foreign substances (mostly food), including microbes and toxins (for example, hot peppers). Chewing hard food can directly hurt soft gingival tissue [82]. The oral environment, with its constant temperature, humidity, and supply of nutrients from food, is a perfect place for microbes to thrive [83]. The hormonal variations during the menstrual cycle are known to alter the properties of oral tissues [63]. Any deficiency in the immunity response to external factors will affect the health status of the oral cavity [84].

Until the onset of genetic microbiome testing, our knowledge of oral microbes was limited. We now have tools for the high-precision identification of not only microbial species, but bacterial strains that become pathogenic due to some genes “borrowed” from other species [85]. But these tools are reserved for those few who understand the technology behind them. Moving this knowledge from its corner to mainstream dentistry and medicine can change the way we treat periodontal disease, allowing a more individualized approach to the treatment. Nowadays, even if we can explore the oral microbiome and link its changes to cancer, pregnancy problems, hypertension, Alzheimer’s disease, etc., there is still no clear path on how we can use this knowledge to treat or prevent periodontal disease and its complications. 

The impact of methodological choices on microbiome analysis results must be thoroughly understood before making any judgments regarding the measured values. One has to take into consideration that even the operating systems of the computers we use in research, like Microsoft Windows, Linux/UNIX, or Apple OS, can induce small but possibly significant changes in the results [15]. Also, there is no consensus regarding the use of a standard procedure to prepare the samples for examination. To keep costs under control and increase efficiency, multiple samples are combined in a single library using a barcode system, but this increases the complexity of measurements and statistical analysis.

Although endogenous hormonal fluctuations appear to exert some influence on the oral microbiome (demonstrated by taxonomic and functional changes in some studies), exogenous factors such as smoking and high sugar consumption could impose stronger, potentially dominant pressures on the structure and diversity of the oral microbiome. This suggests that while the menstrual cycle can induce subtle changes, lifestyle can cause more pronounced alterations, which can mask or interact with the effects related to the estrogen–progesterone cycle. Therefore, interpreting the biological significance of the reported cyclic changes requires a careful examination of the specific methods used in each study.

A few directions for further development can be seen at the end of this review. First and most important is that we need to establish the composition of a healthy oral microbiome. Transversal and longitudinal studies on a larger population group are necessary for this to happen. Due to the high diversity of research protocols we encountered during the writing of the review, no specific recommendations about microbiome testing can be made at this moment. Oral microbiome testing must be simplified to the point where the technology is accessible and reproducible in a normal dental office, and the results can be related to a health problem or status. Additionally, the group of diseases specific to women must be explored directly. 

## Figures and Tables

**Table 1:** Microbiome 16S rRNA gene testing steps.

16S Gene Testing Step	Variable/Activities
Obtaining microbial DNA	Sample
	Preservation
	Storage
	Extracellular DNA depletion
	Human DNA depletion
	Microbial DNA extraction
Library preparation and reading	16S Primer
	PCR amplification
	Barcoding
	Library preparation
	Library reading
Raw data bioinformatics	Computer operating system
	Data analysis platform
	Software
	Eliminate poor-quality readings
	Eliminate PCR artifacts (chimeric sequences)
	Eliminate readings of human DNA
	Merge paired-end readings (if applicable)
	Use a reference genetic database
	Classification algorithms
	Data analysis tools
Microbiome report	Analysis tools
	Data normalization
	Biodiversity (alpha and beta)
	Core microbiome
	Predictive modeling
	Microbial network analysis
	Batch effect

**Table 2:** A list with sample types used in oral microbiome studies and some explanatory details according to Zaura et al. [16].

Sample	Details	
Saliva	Unstimulated saliva	Spit
		Drooling
	Stimulated saliva	Requires gum
		Requires parafilm
	Oral rinse	With mouthwash
		With 0.9% saline
Plaque	Location	Supragingival, pooled or site-specific
		Subgingival
		Interproximal
	Technique	Swabbing
		Scooping
		Paper points
Oral soft tissues swabbing	Location	Palatal swab
		Tonsillar swab
		Buccal swab
		Tongue swab
Dental calculus	Trained researcher in a clinical setting, repeated sample not possible	
Denture surface swab	Specific for edentate individuals	
Mucosal cytobrush	For host–microbe interaction, a high human DNA proportion	
Sub-mucosal biopsy	For host–microbe interaction, an invasive method, a high human DNA proportion	
Negative controls	Test DNA contamination	Sampling blanks (paper points, brushes, tubes)
		DNA extraction kit
		Amplifications kit
Positive controls	Mock community or standard sample	DNA extraction control
		DNA amplification control
		Assessing batch effects

**Table 3:** List of 16S gene primers according to Nagai et al. [19].

Primer	Length bp	Nucleotide Interval
V1–V2	312	27F–338R
V1–V3	492	27F–518R
V3	178	341F–518R
V3–V4	466	341F–806R
V3–V5	567	341F–907R
V4	292	515F–806R
V4–V5	393	515F–907R
V5–V6	317	799F–1115R
V6–V8	434	968F–1401R

## Data Availability

No new data were created or analyzed in this study.

## Abbreviations

The following abbreviations are used in this manuscript:

DNA	deoxyribonucleic acid
RNA	ribonucleic acid
rRNA	ribosomal ribonucleic acid
bp	base pairs, pairs of nucleic acids in DNA and RNA
HOMD	Human Oral Microbiome Database https://v31.homd.org/
PICRUSt	Software Phylogenetic Investigation of Communities by Reconstruction of Unobserved States https://picrust.github.io/picrust/
SILVA	SILVA rRNA database project https://www.arb-silva.de/
PCR	polymerase chain reaction
OTU	operational taxonomic units
ASV	amplionic sequence variants
DADA2	Software https://benjjneb.github.io/dada2/
RDP	Ribosomal Database Project
Greengenes/Greengenes2	Database http://ftp.microbio.me/greengenes_release/
